# Overexpression of annexin gene AnnSp2, enhances drought and salt tolerance through modulation of ABA synthesis and scavenging ROS in tomato

**DOI:** 10.1038/s41598-017-11168-2

**Published:** 2017-09-21

**Authors:** Raina Ijaz, Javeria Ejaz, Shenghua Gao, Tengfei Liu, Muhammad Imtiaz, Zhibiao Ye, Taotao Wang

**Affiliations:** 1 Key Laboratory of Horticultural Plant Biology, Ministry of Education, Huazhong Agricultural University, Wuhan, 430070 China; 2 National Key Laboratory of Crop Genetic Improvement, Huazhong Agricultural University, Wuhan, 430070 China; 3 Microelement Research Center, College of Resources and Environment, Huazhong Agricultural University, Wuhan, 430070 China

## Abstract

Drought and high salinity are two major abiotic stresses that significantly affect agricultural crop productivity worldwide. Annexins are a multigene family that plays an essential role in plant stress responses and various cellular processes. Here, the AnnSp2 gene was cloned from drought-resistant wild tomato (*Solanum pennellii*) and functionally characterized in cultivated tomato. AnnSp2 protein was localized in the nucleus and had higher expression in leave, flower and fruit. It was induced by several phytohormones and some abiotic stresses. Tomato plants overexpressing AnnSp2 had increased tolerance to drought and salt stress, as determined by analysis of various physiological parameters. AnnSp2-transgenic plants were less sensitive to ABA during the seed germination and seedling stages. However, under drought stress, the ABA content significantly increased in the AnnSp2-overexpressing plants, inducing stomatal closure and reducing water loss, which underlay the plants’ enhanced stress tolerance. Furthermore, scavenging reactive oxygen species (ROS), higher total chlorophyll content, lower lipid peroxidation levels, increased peroxidase activities (including APX, CAT and SOD) and higher levels of proline were observed in AnnSp2-overexpressing plants. These results indicate that overexpression of AnnSp2 in transgenic tomato improves salt and drought tolerance through ABA synthesis and the elimination of ROS.

## Introduction

Plants are continuously exposed to many stress conditions, and drought and high salinity have major impacts on plant growth and development. To cope with these unfavorable environmental conditions, plants have adopted various resistance strategies^1^. Stress signaling and transcriptional modulation are important aspects of the complex genetic and biochemical networks that plants use to respond to stress. Ca^2+^ is a prominent signaling molecule for plants under abiotic stresses. Some plant annexins can act as Ca^2+^ transporters in the membrane^2^. Plant annexins are a multigene family of calcium-dependent phospholipid-binding proteins and are found ubiquitously in prokaryotes, fungi, plants and animals^2–4^. The functions of annexin in plants have been inferred largely from gene expression and *in vitro* assays. Different annexins include diverse enzymatic activities, such as peroxidase and ATPase/GTPase activity, as well as calcium channel activity^5,6^.

Some subsequent reports focused on the roles of plant annexins involved in stress signaling^7–9^. Genetic and transgenic approaches have indicated that annexins play an important role in protecting plants from both abiotic and biotic stresses^1,10,11^. Transgenic tobacco plants constitutively expressing the mustard annexin AnnBj1 are more tolerant to different abiotic stress treatments^12^. Overexpression of GhAnn1 in cotton makes the plant more tolerant to different abiotic stress^13^. Plants overexpressing AnnAt1 are more tolerant to drought, and knockout plants are more sensitive to drought than wild-type plants^8^. However, the functions of annexin genes in different species are not always consistent. In contrast to the above findings, mutant plants of annAt1 and annAt4 show tolerance to drought and salt stress in Arabidopsis in a light-dependent manner^1^.

Several reports indicate a role for annexin genes in the actions of plant hormones such as abscisic acid^14^, gibberellic acid^15^, Jasmonic acid^16^, and salicylic acid^8^. ABA is a general regulator of annexin expression in a wide variety of plant species^10,17^. The phytohormone ABA plays a key role in regulating a range of plant physiological processes in response to various stresses^18–21^. Osmotic stress, such as drought and high salinity, dramatically increases the ABA level, which in turn induces the expression of many genes involved in stress responses^22–24^. ABA biosynthesis genes are activated by drought and salt stress^25,26^. Further, biochemical and genetic studies have shown that 9-cis–epoxycarotenoid dioxygenase (NCED) is a key rate-limiting enzyme in ABA biosynthesis^27^ and that over-expression of the NCED gene in tomato and other plants causes abscisic acid (ABA) accumulation and affects stress responses^27–29^. Proline is an important osmolyte that stabilizes macromolecules and membranes in cells exposed to osmotic stress^30,31^. Both drought and salinity can lead to oxidative stress in plant cells, in turn leading to the accumulation of osmoprotective solutes^31^.

Although the first plant annexin was isolated from tomato^32^, only a few functional studies on tomato annexins have been reported^33,34^. Most of them are focused on annexin biochemical properties such as F-actin binding and nucleotide phosphodiesterase activities. Here, we report that over-expression of an annexin gene, AnnSp2, in tomato improved plant resistance related to various abiotic stresses. We investigated detailed stress-related phenotypes and physiological and biochemical parameters under the conditions of water deficit and high salinity. The over-expression of AnnSp2 reduced the plant sensitivity to abscisic acid (ABA) with respect to seed germination and post-germination growth, and the increased ABA content led to smaller stomatal apertures, suggesting that AnnSp2 functions in ABA-mediated drought stress response pathways. Moreover, our results demonstrate that AnnSp2 enhances tolerance to high salinity and oxidative stress by regulating the expression of stress related genes. These data provide new insight into the mechanism by which annexin proteins function in ABA synthesis, which may lead to targeted genetic manipulation of crop plants to improve stress tolerance.

## Results

### Cloning and characterization of AnnSp2 gene

Based on our previous report^35^, a stress-responsive annexin gene, AnnSp2, was cloned and identified from drought-resistant wild tomato (*Solanum pennellii*). The full-length cDNA sequence of AnnSp2 was amplified using gene-specific primers and sequenced (Supplementary Table S1). This cDNA sequence was 1,237 bp and contained a 951-bp open reading frame (ORF), a 146-bp 5′-untranslated region, and a 140-bp 3′-untranslated region. A blast homology search of the tomato genome database (http://solgenomics.net/) showed that the AnnSp2 gene was located in chromosome 4. Sequence analysis predicted that the cloned gene encoded an annexin protein of 316 amino acids, with a molecular weight of 36.2 kDa and an iso-electric point of 5.64.

**Figure 1:**
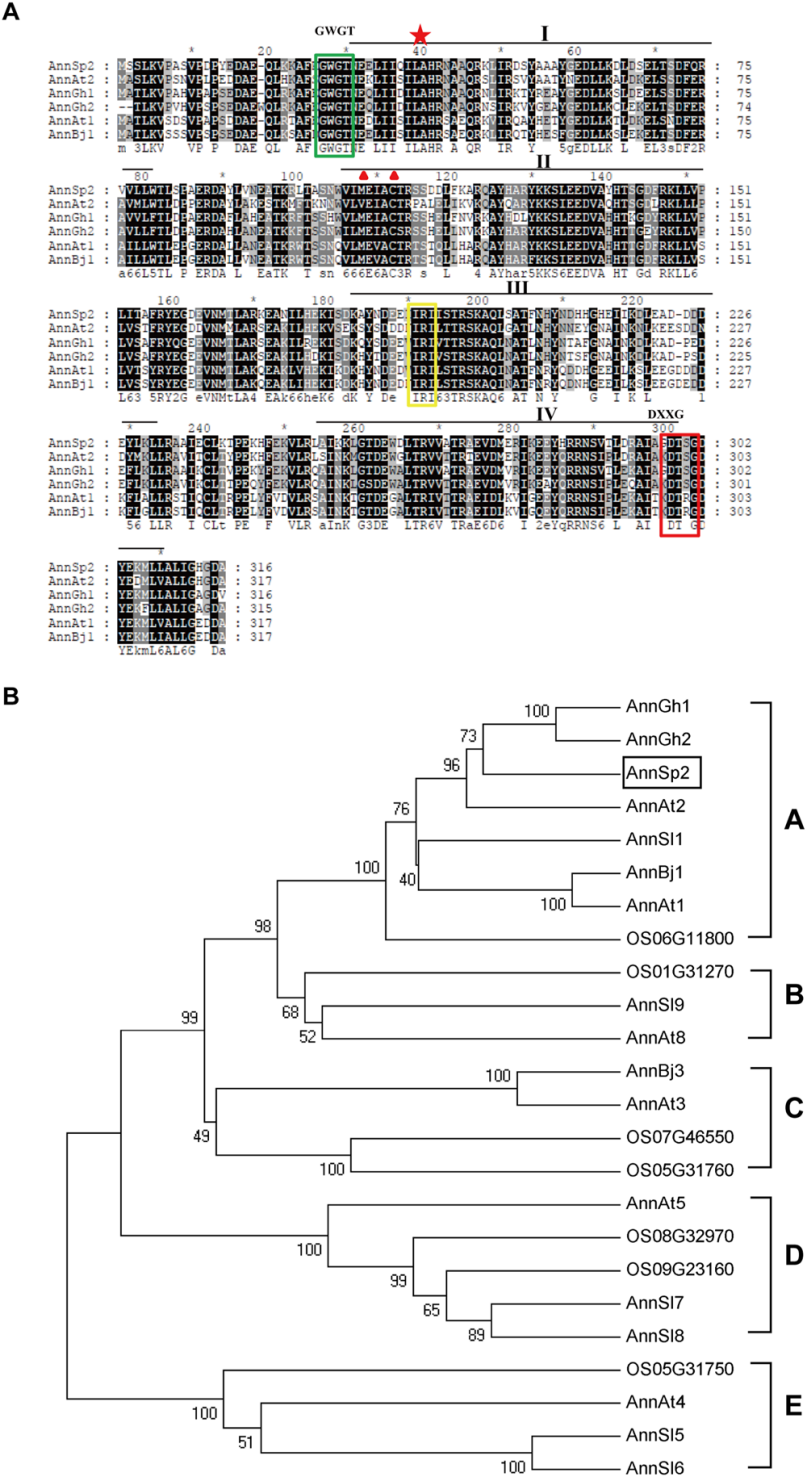
Sequence and phylogenetic analysis of AnnSp2. (**A**) Sequence alignment of AnnSp2 and other predicted annexin protein sequences. The putative annexin protein repeats (I–IV) are shown with black lines. The sequences highlighted are as follows: green box in repeats I, calcium-binding sites; red star, His40 key peroxidase residue; red triangles, putative S3 clusters; yellow box, IRI, actin-binding motif; red box, DXXG, putative GTP-binding motif. (**B**) Phylogenetic analysis of annexin proteins from different plant species. The numbers above or below the branches are the bootstrap values from 1,000 replicates. AnnSp2 is boxed. The accession numbers are as follows: Gh, *Gossypium hirsutum*; AnnGh1 (AAB67993), AnnGh12 (AAB67994), At, *Arabidopsis thaliana*; AnnAt1 (AT1G35720), AnnAt2 (AT5G65020), AnnAt4 (AT2G38750), AnnAt5 (AT1G68090), AnnAt3 (AT2G38760), AnnAt8 (AT5G12380), OS, *Oryza sativa*; OS01G31270, OS06G11800, OS05G31760, OS07G46550, OS08G32970, OS09G23160. Bj, *Brassica juncea*; AnnBj1 (ABD74418), AnnBj3 (ACQ65866), Sl, *Solanum lycopersicum*; AnnSl1 (Solyc04g073990), AnnSl5 (Solyc01g097520), AnnSl5 (Solyc01g097520), AnnSl6 (Solyc01g097550), AnnSl7 (Solyc04g078820), AnnSl8 (Solyc04g008270).

Amino acid sequence alignment of the deduced AnnSp2 with other plant annexins from different plant species revealed high identity (67–76%) with its homologous sequences, including AnnGh1(AAR13288), AnnGh2 (AAB67994), AnnAt2 (AT5G65020), AnnAt1 (AT1G35720) and AnnBj1 (ABD74418). The AnnSp2 and these annexin protein sequences showed that annexin domains are highly conserved and are repeated four times (I–IV) (Fig. 1A). The first repeat contained a Ca^2+^ binding site, and a W residue (position 27) which is present in most plant annexins and maybe important for phospho-lipid-binding. In addition, other sequence signatures suggested to be important for annexin functions were found in the sequence alignment. Such as the His40 residue, IRI motif, and a GTP-binding site (DXXG) (Fig. 1A), these sequence signatures suggest that AnnSp2 has the classical characteristics of annexin family proteins.

To evaluate the evolutionary relationship between AnnSp2 and some known plant annexins; a phylogenetic tree was constructed using MEGA 5.1 based on amino acid sequences of these annexin members (Fig. 1B). Annexin proteins were further divided into five groups (Groups A to E). AnnSp2 belonged to the largest group, group A, which contained eight annexin genes, two from *G. hirsutum*, two from Arabidopsis, one from *Brassica juncea*, two from *S. lycopersicum*, and one from *Oryza sativa*. The AnnSp2 revealed high identity with each other (up to 76% at amino acid level).

### Expression analysis of AnnSp2 and subcellular localization

**Figure 2:**
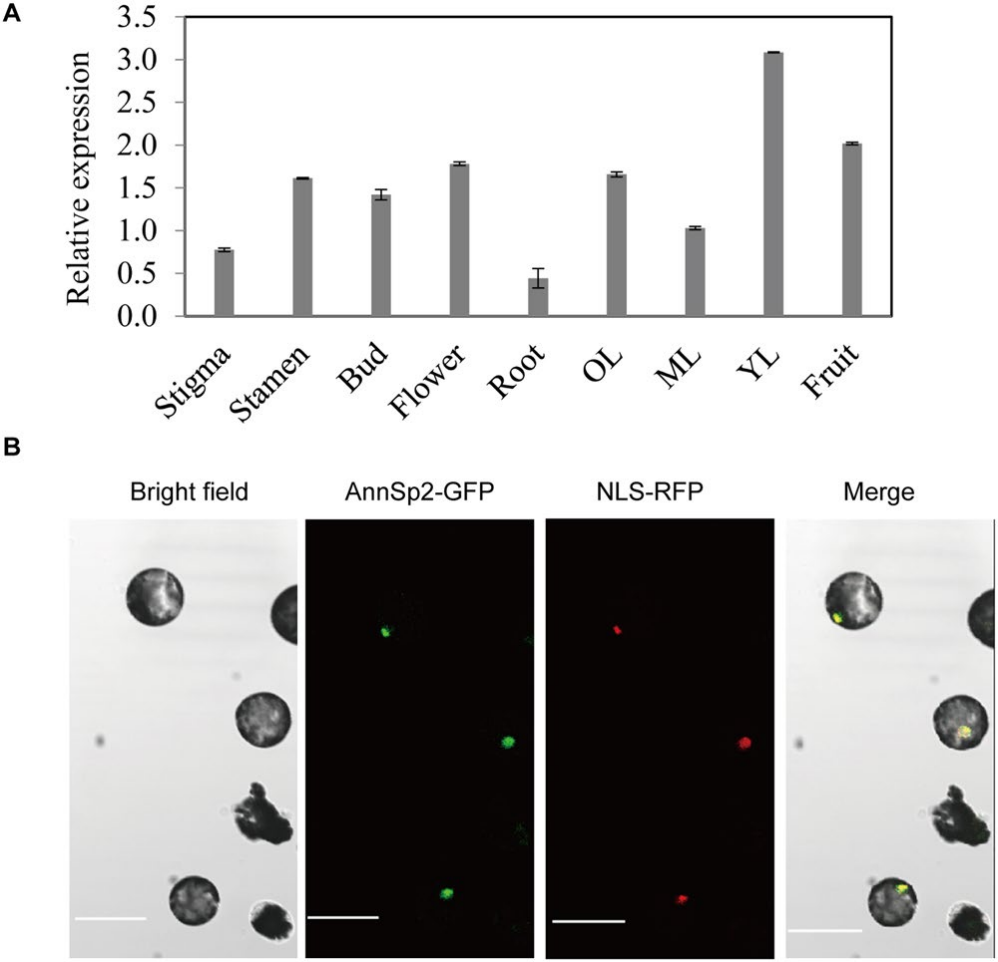
Tissue-specific expression analysis and subcellular localization of AnnSp2 in *N. benthamiana* protoplasts. (**A**) Tissue profiling analysis of AnnSp2 in different organs (Stigma; Stamen; Bud; Flowers; Root; OL, old leaf; ML, mature leaf; YL, young leaf; Fruit) of wild tomato LA0716 (*Solanum pennellii*) using qRT-PCR. (**B**) AnnSp2 localized in the nucleus. The GFP fusion of AnnSp2 was co-expressed with NLS-RFP in *N. benthamiana* protoplasts for 12 h and the images were taken with a confocal microscope. NLS-RFP is a control for nuclear localization. Bar = 50 µm.

The tissue-specific expression pattern of AnnSp2 was investigated in different tomato tissues including stigma, stamen, buds, flowers, roots, stems, leaves and fruits, using qRT-PCR (Fig. 2A). The results showed that AnnSp2 was expressed in all tissues and organs, a relatively higher expression level was observed in the leave, flower and fruit, whereas a relatively lower expression level was observed in the roots.

We also examined AnnSp2-GFP subcellular localization driven by the 35 S promoter, with NLS-RFP as a control for nuclear localization. The AnnSp2-GFP and NLS-RFP of vector constructs were transformed into *N. benthamiana* protoplasts by PEG mediated method. Protein subcellular localization was detected 12 h after transformation using a confocal laser fluorescence microscope (Zeiss LSM510 META, Germany). A strong fluorescence signal from AnnSp2-GFP was detected in the nucleus (Fig. 2B). This result demonstrated that AnnSp2 was a nuclear-localized protein.

**Figure 3:**
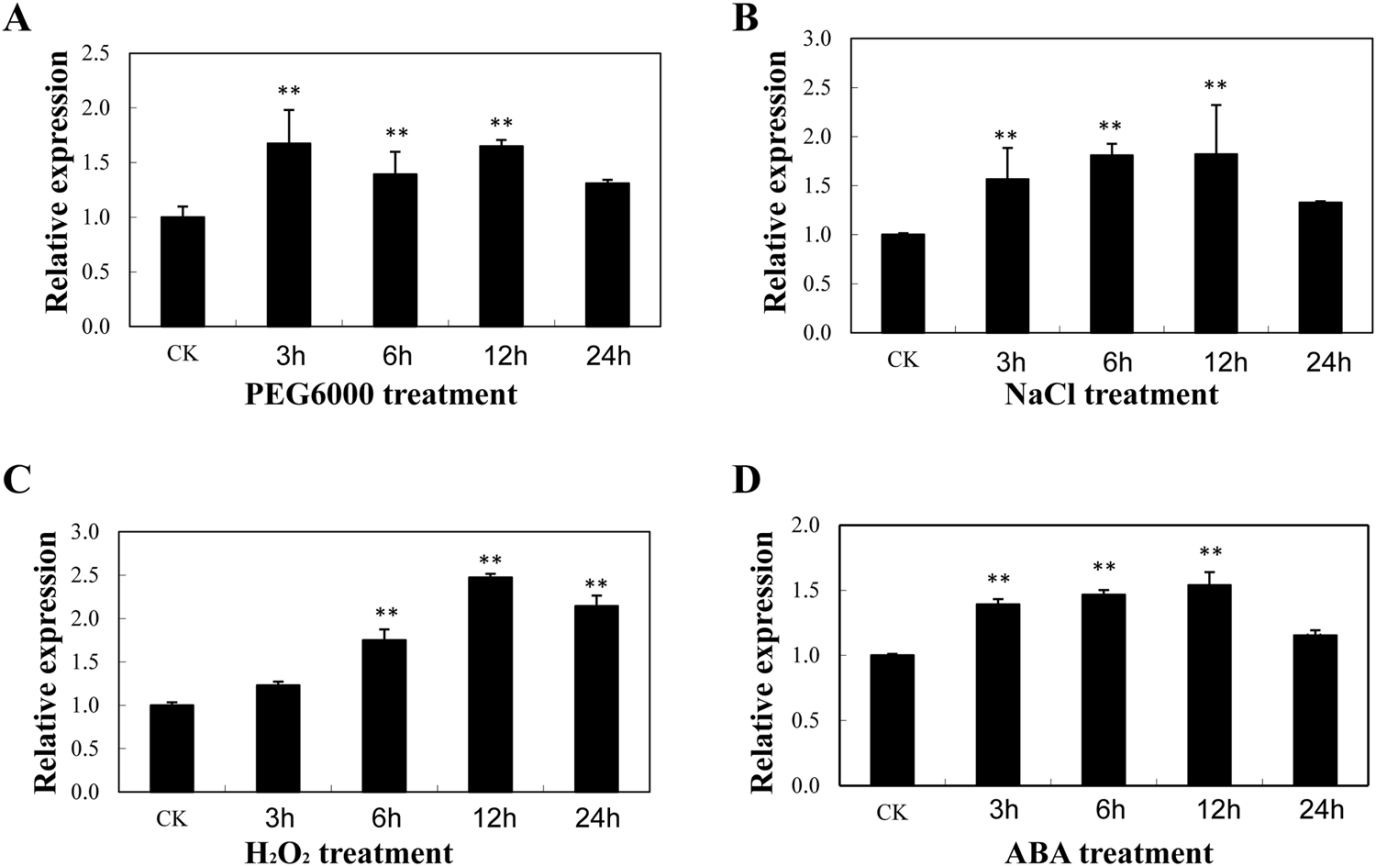
The expression profiles of AnnSp2 in tomato leaves under different abiotic stress conditions. Plants were treated with (**A**) 15% PEG6000, (**B**) 200 mM NaCl, (**C**) 10 mM H_2_O_2_, or (**D**) 100 µM ABA. All samples were collected at the indicated time points (‘CK’ refers to control and ‘h’ refers to hours of treatment). The error bars represent the standard deviations of three biological replicates of each treatment. *p < 0.05, **p < 0.01. The β-actin gene (BT013524) was used as an internal control in the qRT-PCR.

We further examined AnnSp2 expression in response to drought, salt, H_2_O_2_ and ABA treatments using qRT-PCR (Fig. 3). Under drought polyethylene glycol (PEG6000) treatment, AnnSp2 expression was induced after 3 h of treatment, increased to the highest level (1.6-fold induction) at 12 h, and then decreased at 24 h. (Fig. 3A). Under salt stress, the AnnSp2 transcript rapidly increased, reaching its maximum accumulation (1.8-fold induction) at 12 h, and dropped there after (Fig. 3B). The accumulation of hydrogen peroxide (H_2_O_2_) consistently accompanies abiotic stress. Therefore, we measured the effect of H_2_O_2_ on AnnSp2 transcription. Its expression gradually increased after 3 h and peaked at 12 h (Fig. 3C). Following ABA treatment, AnnSp2 was up-regulated, with a peak at 12 h (1.5-fold induction) (Fig. 3D). These findings suggest that AnnSp2 might play a role in the response to multiple abiotic stresses.

We next cloned and analyzed the promoter of AnnSp2. Using the PlantCARE databases, some *cis*-acting regulatory elements associated with the stress response were predicted in this promoter region (Supplementary Fig. S1). Specifically, many abiotic stress-responsive elements involved in the light response and the phytohormone response, such as ABA, GA3, and abiotic stress (e.g., MYB binding site and heat stress response), were identified in the AnnSp2 promoter region. In addition, a number of tissue-specific and development-related elements were found. These results suggest that AnnSp2 maybe play a role in the plant response to environmental stresses and in development.

### Overexpression of AnnSp2 in transgenic tomato enhances salinity and drought tolerance

Transgenic plants overexpressing AnnSp2 were generated to evaluate the function of AnnSp2. A total of 14 kanamycin-resistant transgenic lines were obtained and confirmed using cauliflower mosaic virus (CaMV) 35 S forward and gene-specific reverse primers (Supplementary Table S1). Their transcript level was determined by qRT-PCR and protein accumulation was detected by western blot using AnnSp2-specific antibody (Supplementary Fig. S2). Three homozygous T3 lines, OE1 (3#), OE2 (8#) and OE3 (12#), with relatively high expression of transcript and protein were selected to do further assays.

**Figure 4:**
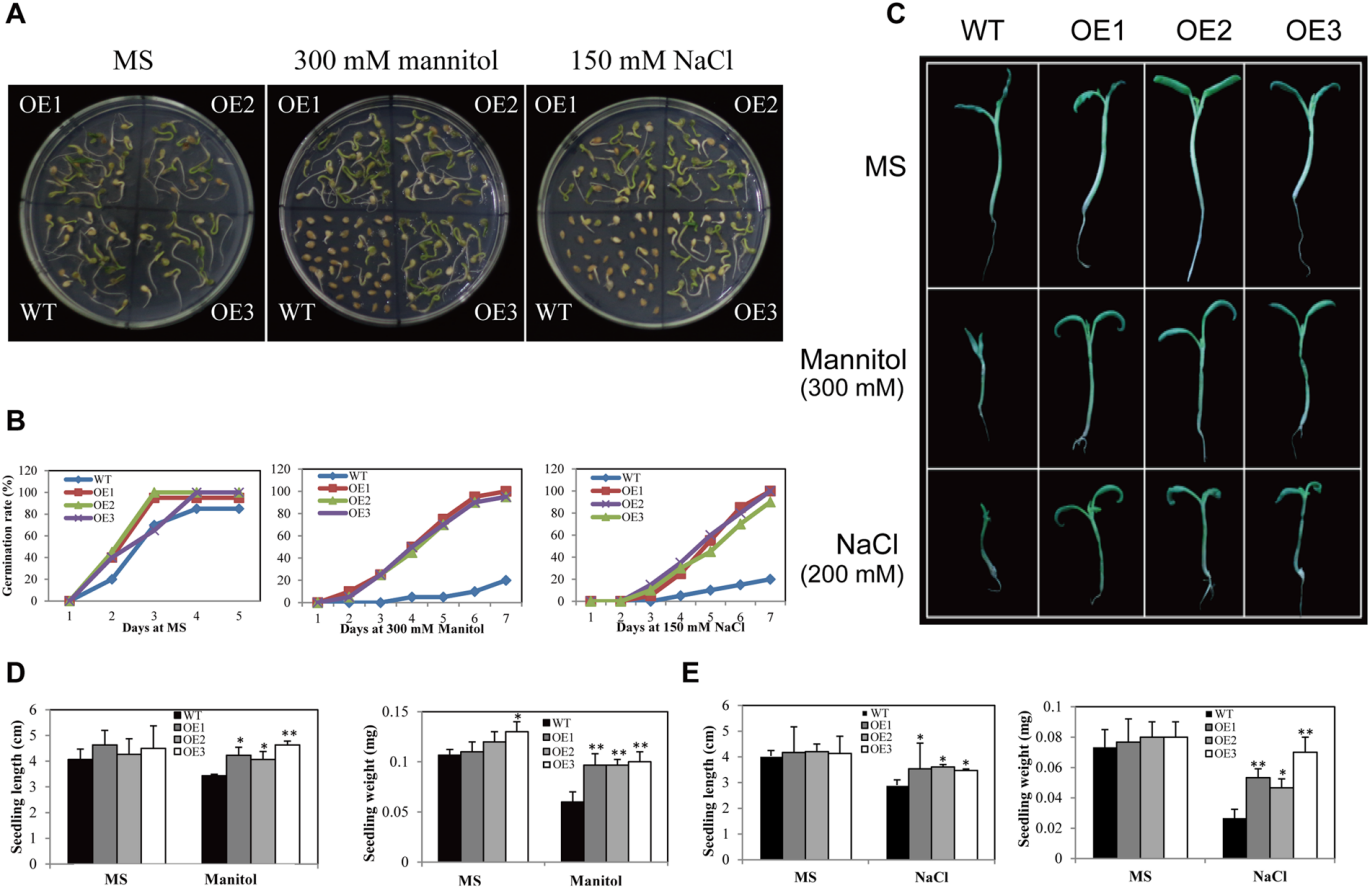
Analysis of germination and seedling elongation in WT and transgenic tomato in response to drought and salt stress. (**A**) Germination of AnnSp2-transgenic seeds after treatment with 300 mM mannitol or 150 mM NaCl. (**B**) Statistical analysis of the seed germination rate. (**C**) Phenotypes and seedling length of WT and transgenic lines. Seedling lengths and weights of AnnSp2-transgenic and WT plants after treatment with 300 mM mannitol (**D**), 200 mM NaCl (**E**), or no stress as a control. The seedlings were grown in MS medium and 200 mM NaCl. The data shown are the mean ± SE (n = 6). *p < 0.05, **p < 0.01, transgenic vs. wild-type.

To determine whether AnnSp2 is involved in abiotic stress tolerance, drought and salt stresses with mannitol (300 mM) or NaCl (150 mM) were applied to the seeds of transgenic OE lines and wild-type (WT) in MS medium. First, we tested the seed germination rate of OEs and WT upon two abiotic stresses. There was no obvious difference in germination rate between the WT and OE plants in MS medium. However, in the presence of mannitol or NaCl, the germination rate of WT plants decreased by approximately 52% to 61% compared with the OE plants (Fig. 4A,B). Second, the seedling length and seedling weight of the WT and OE lines under drought and salt stress were calculated (Fig. 4C–E). Upon mannitol stress, the seedling length decreased by 16% for WT and by approximately 10% for the OE lines, whereas seedling weight decreased by 47% for WT and only 12–23% for the OE lines (Fig. 4D). For salt stress treatment, slightly difference was observed between WT and OE lines in terms of seedling length, whereas seedling weight decreased by 50% for WT and by 15–41% for the three AnnSp2 OE lines (Fig. 4E). Overall, the transgenic lines overexpressing AnnSp2 experienced less growth inhibition compared with WT plants under drought and salt stresses, especially in terms of seedling weight.

**Figure 5:**
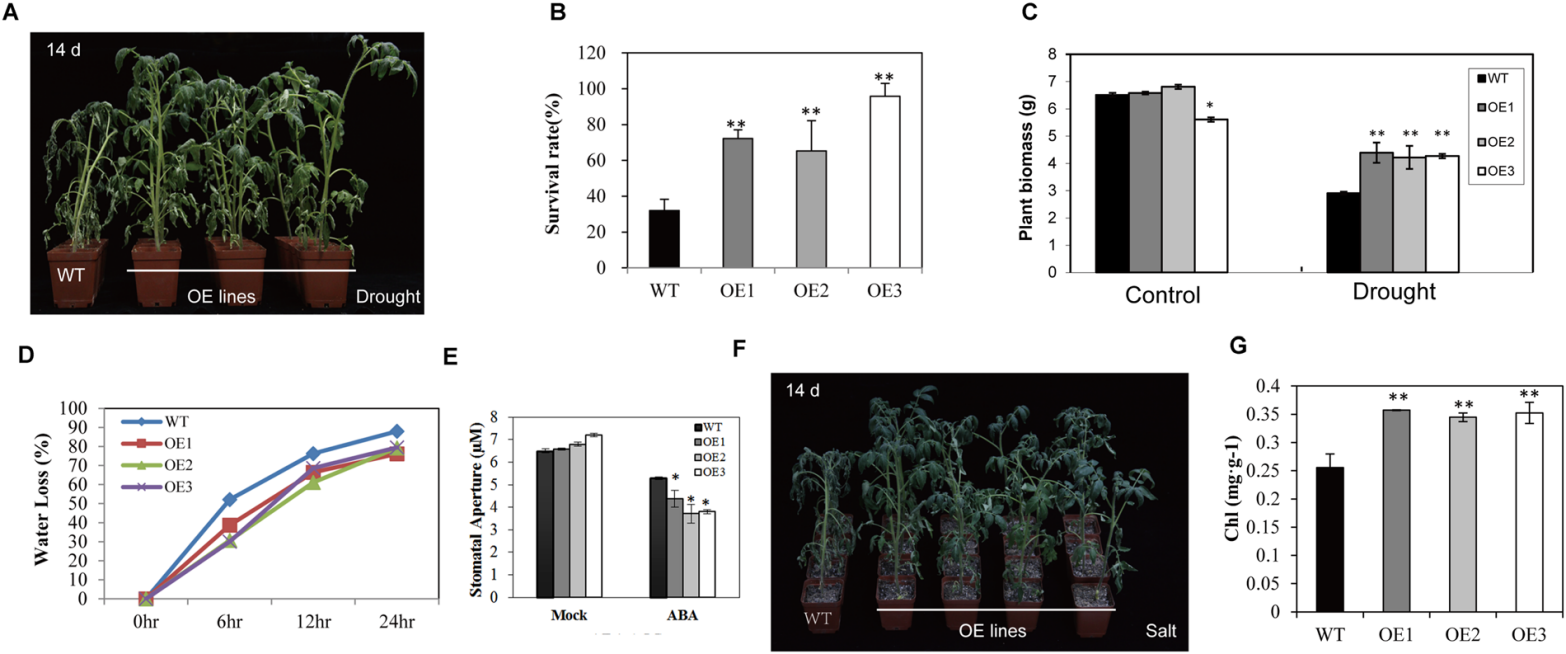
AnnSp2 overexpression enhances drought and salt stress tolerance in tomato. (**A)** Drought tolerance test for AnnSp2-overexpressing and WT plants. Drought stress was imposed by withholding water for 14 days. The phenotypes under drought conditions are shown. (**B**) Survival rates of the plants after re-watering of the OE and WT plants. The SE values (error bars) were calculated from three independent experiments (n ≥ 25 for each experiment). (**C**) Plant biomass on drought-stressed transgenic and WT plants for two weeks. (**D**) Water loss rate of the detached leaves from WT and AnnSp2-transgenic plants under drought stress. (**E**) Statistical analysis of the stomatal aperture before and after ABA treatment. (**F**) Phenotype of AnnSlp2-OE plants exposed to 14 d salt stress. (**G**) Quantification of the chlorophyll content in plant in the salt-treated plants relative to that of the untreated plants in three independent experiments (n > 20). The data shown are the mean ± SE (n = 3). *p < 0.05, **p < 0.01, transgenic vs. wild-type.

We next tested the performance of the adult transgenic plants under drought and salt stress tolerance. Four-week-old soil-grown transgenic and wild-type plants were exposed to drought stress by continuous withholding of water for 7–14 days and observed for phenotypic differences. After withholding of water 7 days, the WT plants showed obvious wilting symptoms, whereas the transgenic plants displayed less wilting. WT plants became more withered after a two-week exposure to drought compared with the transgenic plants (Fig. 5A). After watering was resumed, 87% of the overexpressing lines survived, compared with 29% of the WT plants (Fig. 5B). The biomass of the WT plants was decreased by approximately 59%, whereas that of the transgenic plants was decreased by only approximately 25% (Fig. 5C). We next performed a water loss assay with detached leaves. The rate of water loss from the WT plants was faster than that from the AnnSp2-transgenic plants (Fig. 5D). ABA-mediated stomatal closure regulates the level of transpiration under water-deficit conditions. Thus, we also tested whether the reduction of water loss in adult transgenic tomato would affect the sensitivity of guard cells to ABA treatment. Under normal conditions, the wild-type and overexpressing plants showed the same level of stomatal apertures. However, a significant reduction of stomatal apertures in OE plants was observed compared to that of WT plants after ABA treatment (Fig. 5E).

For the salt tolerance assay, six-week-old AnnSp2 OE and WT plants were irrigated with 200 mM NaCl solution for 14 d. The growth of the WT plants was severely affected by salinity, but not that of the OE plants. Furthermore, the signs of stress were more severe in the WT plants, which displayed serious chlorosis and wilting (Fig. 5F). The total Chl content, which reflects the level of chlorosis, was significantly higher in the transgenic plants upon NaCl stress (Fig. 5G).

### Overexpression of AnnSp2 in tomato alleviates sensitivity to ABA according to seed germination and post-germination growth assays

**Figure 6:**
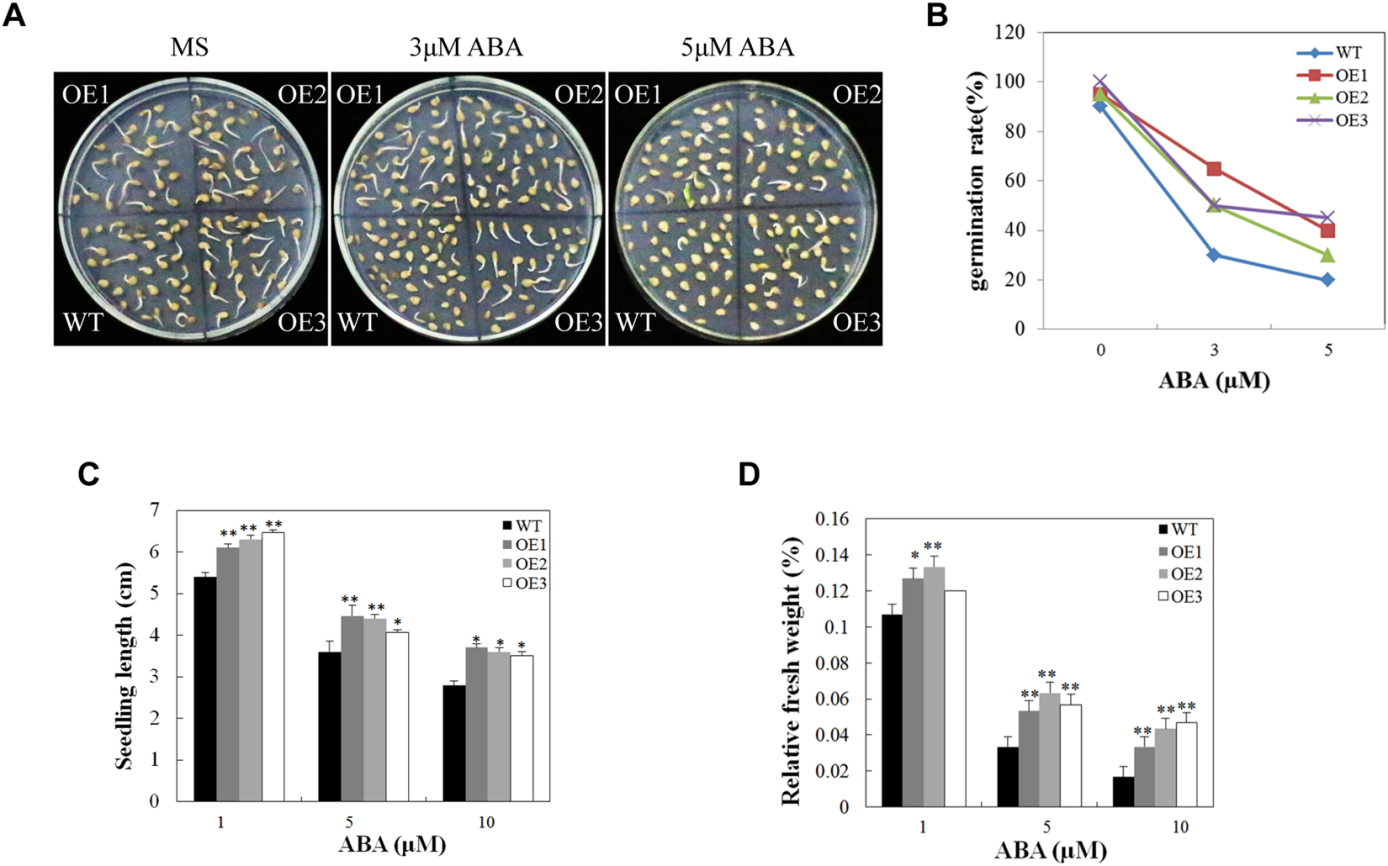
The overexpression of AnnSp2 could impair ABA sensitivity in tomato plants. **(A** and **B)** Comparison of seed germination between the WT and OE lines exposed to 0, 3, 5 or μM ABA. The data represent the means ± SE of three independent experiments, with 30 seeds per genotype and experiment. (**C**) Measurement of seedlings after ABA treatment. Three independent experiments were performed with similar results. Each value represents the mean ± SE of at least 30 seedlings from three independent experiments. (**D**) Measurement of seedling fresh weight after ABA treatment. Sterisk indicate significant differences *p < 0.05, **p < 0.01, transgenic vs. wild-type.

The increased expression of AnnSp2 in response to exogenous ABA suggested that AnnSp2 might play a role in ABA synthesis. Thus, the biological function of AnnSp2 in response to ABA was investigated. The seeds of the three overexpressing lines and wild-type were germinated on MS medium. After 3d, we investigated the germination rates of seeds. The OE lines and WT showed similar germination rates under normal conditions. However, the germination rate is reduced along with the increase of ABA concentration, when supplemented with 5 µM ABA in medium, the germination rate was approximately 40% in OE lines compared with approximately 20% in the WT (Fig. 6A,B).

The WT and AnnSp2-transgenic plants were also measured for their responses to abscisic acid (ABA) during the post-germinative growth stage. The seeds were germinated on MS medium for 3d and then transferred to medium supplemented with ABA at 1, 5 or 10 μM. The seedling length and weight decreased as the increasing of ABA concentration in both WT and OE plants. However, WT plants exhibited a greater reduction in the seedling length and weight compared with OE lines. Overall, the seedling length was decreased by 52% for WT and only by 27–34% for the OE lines, whereas seedling weight decreased by 56% for WT and 30–41% for the OE lines (Fig. 6C,D). Together, these data demonstrate that AnnSp2 could partially impair the ABA sensitivity in tomato plants during germination and seedlings stage.

### Expression of stress-responsive genes in AnnSp2-transgenic plants

**Figure 7:**
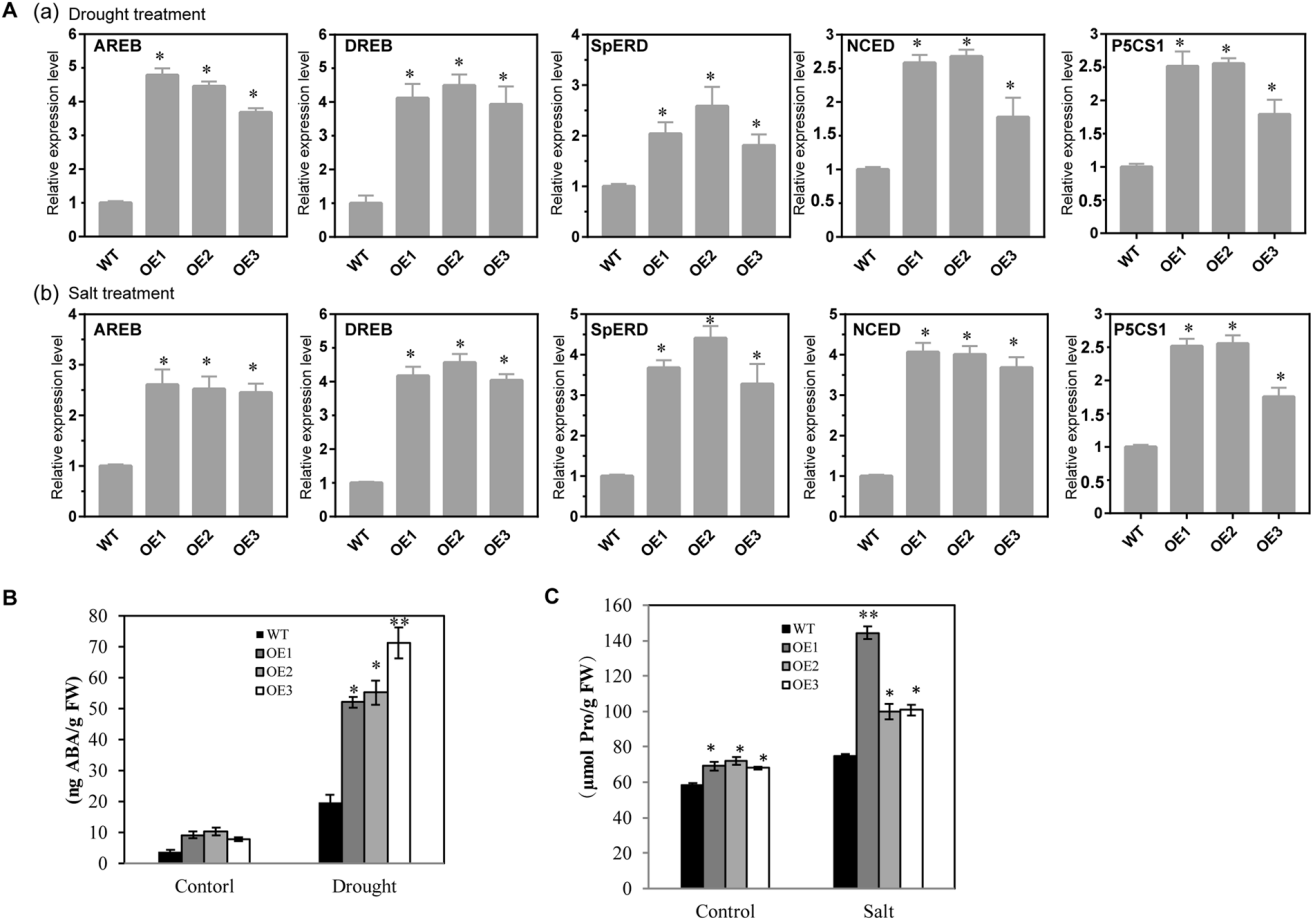
Expression of stress-responsive genes and production of ABA and proline in AnnSp2-transgenic and WT plants in response to drought and salt stress. (**A**) qRT-PCR analysis of stress-responsive genes in WT and transgenic plants upon drought (a) and salt treatment (b). (**B**) Endogenous ABA content in the leaves of AnnSp2-transgenic plants and WT plants under normal and water-deficit conditions. (**C**) Proline content upon salt. Proline was quantified spectrophotometrically. The data shown are the mean ± SE (n = 3). ANOVA was performed to determine significant differences. *p < 0.05, **p < 0.01, transgenic vs. wild-type.

We performed qRT-PCR to examine whether the expression of some stress-responsive genes might be altered in the transgenic plants under drought and salt stress, such as early responsive to dehydration (ERD), dehydration-responsive element binding (DREB) and ABA-responsive element binding (AREB). These genes were induced in transgenic plants compared with WT under the same treatment conditions (Fig. 7A). In addition, we assessed the expression of 9-cis–epoxycarotenoid dioxygenase (NCED) and ∆1-pyrroline-5-carboxylate synthase (P5CS1), which encode a key gene in ABA and proline biosynthesis under drought and salt stress respectively. The expression of these two genes was higher in transgenic plants compared with WT plants under drought treatment in some extent (Fig. 7A). To test whether altered expression of NCED and P5CS1 led to changes in their product accumulation in plants, we then measured the endogenous ABA and proline contents (Fig. 7B and C). The accumulation of ABA and proline was already higher in AnnSp2-transgenic lines than in wild-type under control conditions, but drought and salt treatment caused greater accumulation of ABA and proline in OE plants than in the WT plants, consistent with increased expression of NCED and P5CS1 in AnnSp2-transgenic plants. These results suggest that overexpressing AnnSp2 may play an important role in regulating gene expression in response to drought and salt stress.

### Overexpression of AnnSp2 enhances the tolerance to oxidative stress by increasing ROS-scavenging ability under drought and salt stress

**Figure 8:**
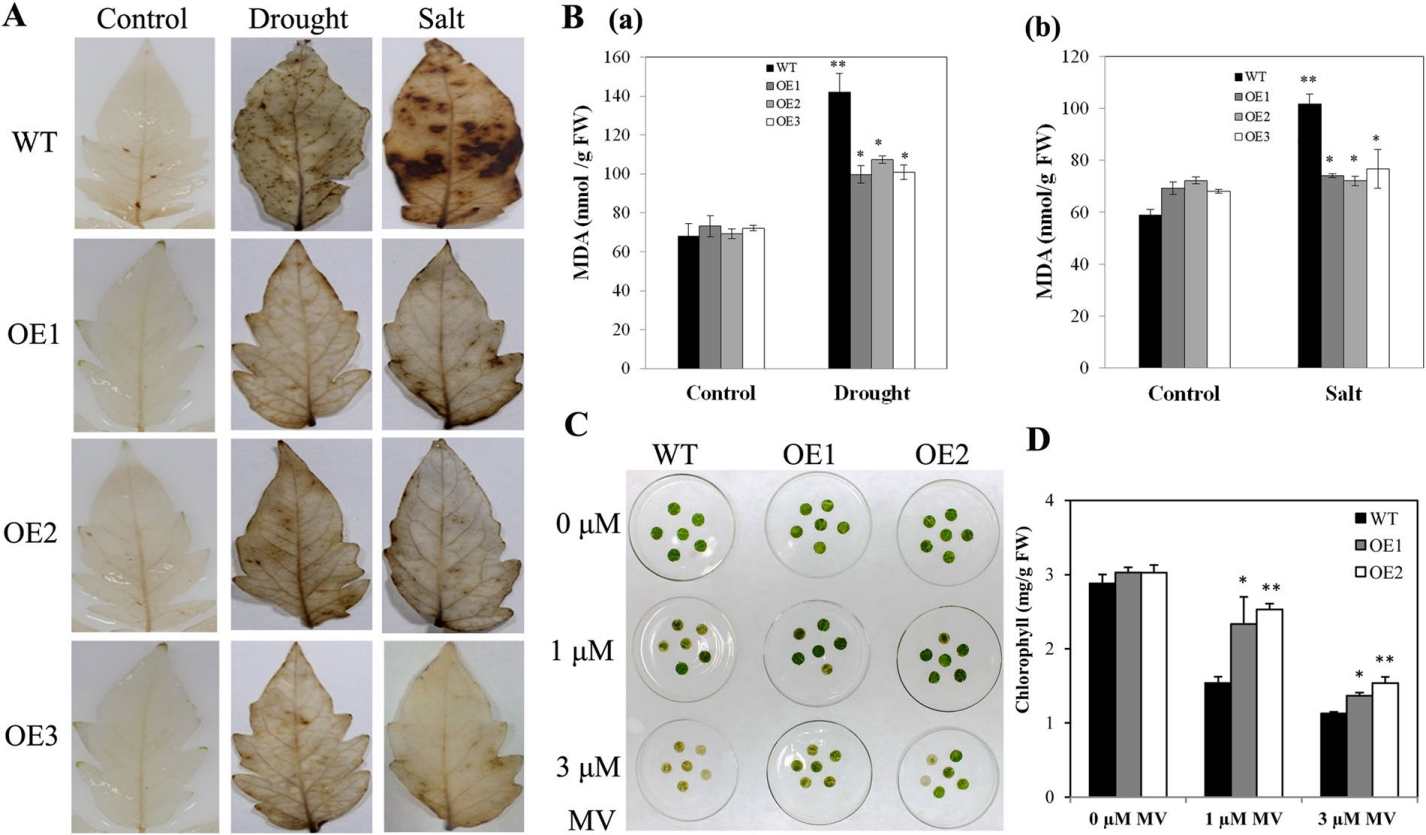
ROS accumulation and oxidative stress tolerance in AnnSp2-transgenic plants under drought and salt stress conditions. (**A**) H_2_O_2_ accumulation visualized by DAB on day 8 during drought or salt treatment. (**B**) MDA content in the WT and OE lines under drought (a) or salt (b) stress. (**C**) Leaf discs obtained from 8-week-old WT and OE seedlings were incubated in different concentrations of MV for 72 h before the photos were taken. (**D**) Chlorophyll content of leaf discs while leaf discs were floating in water served as a control. The data shown are the mean ± nSE (n = 3). ANOVA was performed to determine significant differences. *p < 0.05, **p < 0.01, transgenic vs. wild-type.

It is likely that drought and high salinity cause oxidative damage due to the production of reactive oxygen species (ROS)^8,36^. We tested whether AnnSp2 regulates ROS levels in response to drought and salt stress. H_2_O_2_ and O^−2^ are two prominent ROS species that are involved in abiotic stress signaling. Therefore, the oxidative burst was determined using diaminobenzidine (DAB) staining under normal and stress conditions using detached leaves. As shown in Fig. 8A, under normal conditions, no obvious difference was observed between the OE and WT lines. After exposure to drought and salt stress, DAB staining showed the WT plants accumulated more H_2_O_2_ and O^−2^ than the transgenic plants, indicating that AnnSp2 may enhance the ROS-scavenging capacity under drought and salt stress in transgenic tomato plants.

This result was further confirmed by malonaldehyde (MDA) content, which reflects membrane injury and membrane lipid peroxidation. Interestingly, the basal level of MDA was lower in WT than in transgenic plants under normal conditions (Fig. 8B). However, the overexpressing lines and wild-type plants showed significantly different following two stress treatments. The MDA level in the 3 OE lines was 26–28% lower than WT in response to drought and salt stress treatments. This distinctive change in the content of MDA indicates that AnnSp2 can alleviate cell membrane injury after exposure to stress conditions.

The potential role of AnnSp2 in oxidative stress was further investigated in response to MV in transgenic plants. We used methyl viologen (MV), an herbicide that induces oxidative stress and inhibits electron transport during photosynthesis through ROS production^37^. Leaf disks from transgenic and WT plants were incubated in a range of MV concentrations (0–3 μM) for 72 h. A greater extent of bleaching or chlorosis was observed in WT leaf disks than in the OE lines (Fig. 8C). The total chlorophyll (Chl) content further confirmed the oxidative damage between the transgenic and WT plants (Fig. 8D).

**Figure 9:**
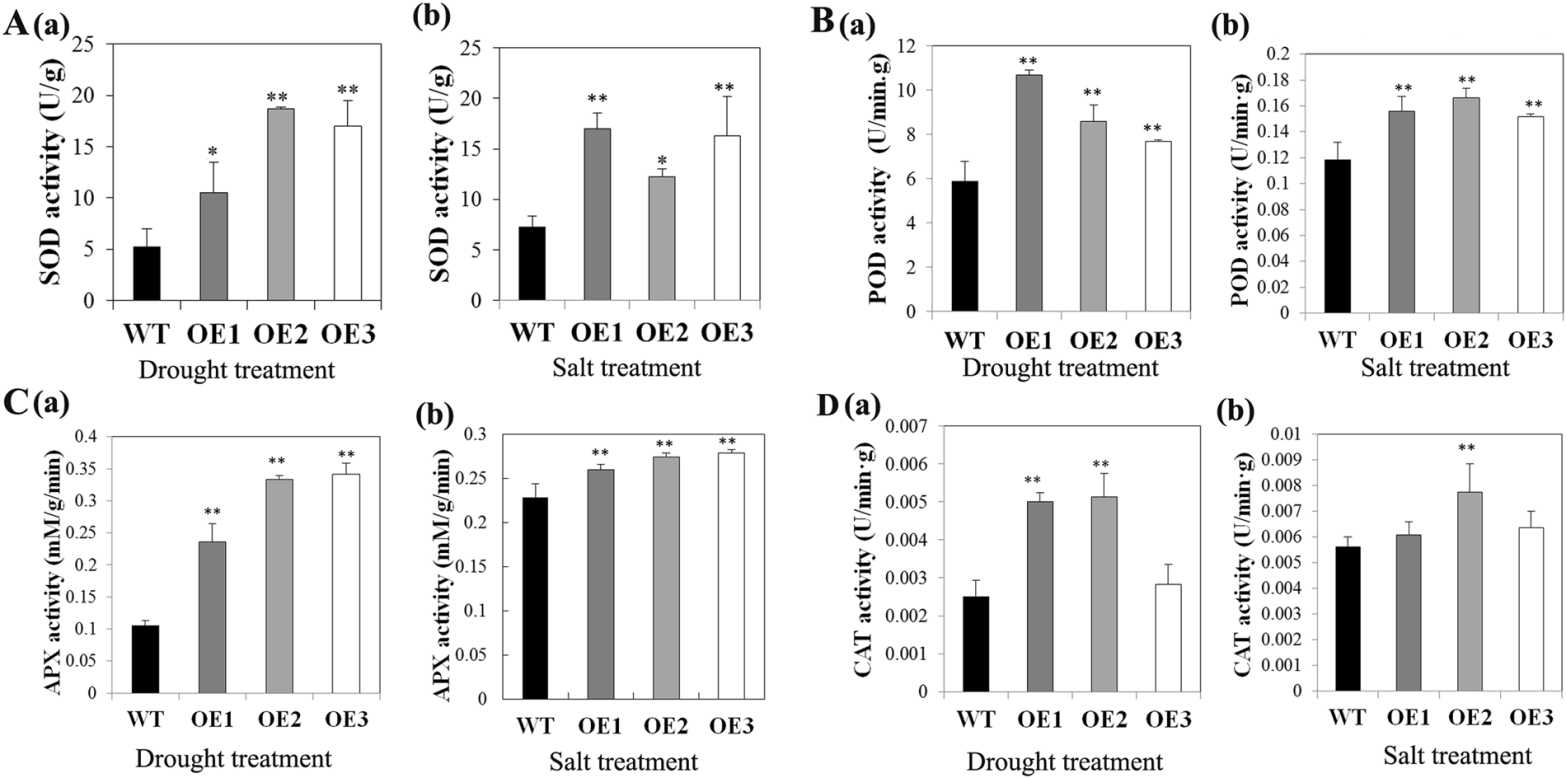
Antioxidant enzyme activity in WT and AnnSp2-transgenic plants under drought and salt stress conditions. (**A**–**D**) Activities of SOD, POD, CAT and APX, respectively. The values represent the means ± SE of three independent experiments. ANOVA was performed to determine significant differences. *p < 0.05, **p < 0.01, transgenic vs. WT.

Moreover, the total activity of antioxidant enzymes like superoxide dismutase (SOD), peroxidase (POD), catalase (CAT) and ascorbate peroxidase (APX) was significantly increased in transgenic lines compared with WT plants in response to both drought and salt stress (Fig. 9A–D). There were no significant differences in SOD activity between WT and transgenic plants under normal conditions. However, under drought stress, there was a striking elevation in the transgenic plants compared to in WT, with a maximum level of 3-fold reached in OE2 line (Fig. 9A). Similarly, transgenic plants displayed markedly higher levels of SOD activity with NaCl treatment (Fig. 9A). Under drought treatment, the POD activity in the transgenic lines increased up 1.92-fold, and the greatest increase was observed in transgenic line OE2, with a 1.6-fold increase in response to salt treatment (Fig. 9B). The APX activity in the transgenic plants increased as much as 3-fold, in OE3, compared with WT in response to drought. Its activity also significantly increased in response to salt stress (Fig. 9C). The CAT activity in the transgenic lines OE1 and OE2 was 2–3-fold higher than WT exposed to drought treatment (Fig. 9D).

## Discussion

Plant annexins play key roles in responses to abiotic stresses. However, the involvement and significance of annexins in the response to stress in tomato remain largely unknown^15^. We previously reported that AnnSp2 is associated with the stress-responsive protein SpUSP^35^. The molecular and physiological mechanisms of AnnSp2 action were unknown, so we cloned and overexpressed it in tomato to explore the function of the AnnSp2 gene in the abiotic stresses in tomato. Annexin genes are expressed in most tissues and organs^2^, and their expression is regulated by developmental processes and induced by diverse types of environmental stress^38^. The expression of nine genes encoding putative annexin proteins (AnnSl1-AnnSl9) is induced by some abiotics and plant hormones^15^. In the present study, expression profiling revealed differential accumulation of AnnSp2 transcript in all tissues (Fig. 2A) which, suggests that it also plays a role in normal plant growth. In spite of high sequence similarities between AnnSp2 and AnnAt2, the expression pattern of AnnSp2 is quite different. AnnAt2 is specifically highly expressed in the roots and less so in stem and flowers^39^. Here, AnnSp2 was more highly expressed in leaves, flower and fruit, lower in root. These results suggest that the expression patterns of annexins may be varied in different species or depend on both developmental stage and environment condition.

Our results indicated that AnnSp2 is a nuclear localized protein. It is interesting about the subcellular localization, because most annexins are PM localization, Similar nuclear localization studies has been reported in pea and alfalfa cells for annexin genes^40,41^, we also found nuclear localization signal (NLS) in the AnnSp2 sequence, but the nuclear localization function is unknown largely. it maybe interact with Ca^2+^ or some potential binding partner to activated transcription, replication, and/or nuclear membrane processes^40^. Further studies are needed to determine its function of nuclear localization in plants.

Phytohormones have different functions in growth, development, and environmental adaptation of plants. The induced expression of annexin genes by abscisic acid (ABA) or stress treatments has been reported in Arabidopsis^10,42^, alfalfa^41^, Indian mustard^43,44^, tomato^15^ and cotton^13^. We also found that AnnSp2 was up-regulated in tomato in response to ABA (Fig. 3). ABA mediates the core signaling network in the plant abiotic stress response^20^, and we found that AnnSp2 alleviated ABA sensitivity in tomato in the germination and seedling stages (Fig. 6). In contrast, the transgenic plants overexpressing annexin in Arabidopsis showed sensitivity to ABA, salt and mannitol in seed germination^1,10^. However, we found that under mannitol and NaCl exposure, the germination and seedling growth of AnnSp2-transgenic plants were significantly increased compared with wild-type plants (Fig. 4), which was similar to previous findings for annexin genes from Arabidopsis seedlings under different abiotic stresses^38^. All of the above results indicate that AnnSp2 enhances tolerance to drought and salinity compared to WT, dependent upon the presence of ABA.

Drought and salt stress can induce ABA accumulation as a result of the activation of biosynthesis of ABA and accumulation of osmoprotectants^18^. ABA treatment stimulates stomatal closure in leaves and enhances resistance to drought in myb96-overexpressing Arabidopsis^45^. In the present study, the increased ABA content and less stomatal aperture, suggesting that AnnSp2 has a function in regulating stomatal opening and thus improves drought resistance by ABA signaling. Similar results were reported for its interaction protein SpUSP^35^. This is reasonable because these two proteins are interacted, they should use similar mechanism to regulate the abiotic tolerance. During drought and salt stress, ABA affects the water balance and cellular dehydration tolerance through guard cell regulation has been reported^45–47^.

Drought and salinity impose osmotic stress, which leads to the production of ROS^48^. ROS, which mainly consist of O_2_^−^ and H_2_O_2_, act as signaling molecules for the initiation of cellular responses to various stresses. High concentrations of ROS ultimately result in oxidative stress and cell damage in the absence of rapid scavenging by antioxidant enzymes^49,50^. ROS plays dual roles in cell extensibility and frequently acts to elevate cytosolic free Ca^2+^ by increasing calcium flux, and cytoplasmic Ca^+2^ is important in guard cell ABA signal transduction^51,52^. Annexins play important roles in ROS-related signaling pathways. AtAnn1 is involved in H_2_O_2_-activated Ca^+2^ fluxes^8^. ROS-regulated Ca^+2^ transport protein GhAnn1 modifies the accumulation of H_2_O_2_ and cytosolic Ca^2+^ in cotton root cells under drought and salt stresses^13^. There are several other early signaling events in which these annexins could function during stress responses to result in detoxification of ROS, including acting on calcium channels directly, regulating enzyme activities, or participating in lipid microdomain formation. In the present study, drought and salt stress in transgenic tomato plants overexpressing AnnSp2 caused less accumulation of hydrogen peroxide, as indicated by DAB staining and the lower MDA level, implying that the presence of AnnSp2 in the transgenic plants protected cellular membranes by inhibiting lipid peroxidation during stress treatments, possibly via detoxification of ROS (Fig. 8). This conclusion is consistent with that of ref.^53^, who reported that an aldose/aldehyde reductase protected against lipid peroxidation in response to abiotic stresses.

In addition, membrane damage and cell membrane instability induced by oxidative stress have been used as criteria to assess the degree of salt and drought tolerance of plants^54,55^. Plants have developed several antioxidative strategies to eliminate these toxic compounds. The up-regulation of the activities of ROS-scavenging enzymes protects plant cells against oxidative damage due to ROS. Antioxidation is an important component of multiple abiotic stresses and includes SOD and ascorbate peroxidase (APX). The enhancement of antioxidant defense in plants can increase tolerance to various stress factors^50,56,57^. Here, the activities of antioxidant enzymes, including superoxide dismutase (SOD), peroxidase (POD), catalase (CAT) and ascorbate peroxidase (APX), in the AnnSp2-OE plants were higher than those in the WT plants under conditions of drought and high salinity (Fig. 9A–D), which contributed to the enhanced tolerance to drought and salt stresses in the transgenic plants. Furthermore, the increased tolerance of AnnSp2 after exposing the plants to MV confirmed the involvement of AnnSp2 in ROS signaling.

Our current and previous findings^35^ lead us to propose that AnnSp2-overexpressing tomato exhibits important physiological functions in the drought and salt responses through the regulation of ABA and ROS. The mechanism of AnnSp2 in governing the plant response to drought and salt stress show a high degree of similarity, most probably because salt stress is often accompanied by drought stress. Further with supporting our conclusion,^18^reported that both stresses induce water deprivation through ABA- dependent and ABA-independent pathways. However, the more specific molecular mechanism still needs to be tested in future experiments.

## Materials and Methods

### AnnSp2 cloning and sequence analysis

Total RNA was extracted from the leaves of the wild tomato species *Solanum pennellii* LA0716 using Trizol reagent (Invitrogen, USA). Reverse transcription PCR (RT-PCR) was performed using a reverse transcription kit (Toyobo, Japan). The tomato annexin2 gene (Sopen04g025030.1, referred to AnnSp2) was amplified from the cDNA of *S. pennellii*, using the primers AnnSp2-F and AnnSp2-R (Supplementary Table S1). The amplified PCR fragment was cloned into pMD18-T vector and transformed into DH5α *E. coli* cells, and the correct clone was selected after sequencing. A phylogenetic analysis was conducted as previously described by ref.^15^. The *cis-*acting regulatory elements in the promoter region were analyzed using the PlantCARE^58^ and PLACE databases^59^.

### Plant growth and stress treatments

For gene expression profiling analysis, 2-month-old tomato plants (LA0716, *S*. *pennellii*) were grown under greenhouse conditions at 25 °C and a 14-h light/10-h dark cycle and subjected to various stress or hormone treatments. For the drought and salinity treatments, plant roots were placed in a solution containing 15% PEG6000 (w/v) or 200 mM NaCl, respectively. For hormone treatments and oxidative stress, 100 µM ABA and 10 mM H_2_O_2_ were sprayed directly onto tomato plants. After each treatment, leaves from different plants (three biological replicates) were collected at appropriate times, immediately frozen in liquid nitrogen and stored at −80 °C for RNA extraction. Each treatment was repeated at least twice.

### Vector construction and plant transformation

To overexpress AnnSp2 in tomato, the pBI121 vector containing the target gene was introduced into the *Agrobacterium tumefaciens* strain C58 through electroporation, plant transformation was performed with a cultivated tomato (*Solanum lycopersicum*) Alisa Craig (AC57), the transformation was performed using the standard leaf disc method, as previously described by^60^. The transformants were selected using kanamycin resistance (50 µg ml^−1^), and the presence of the transgene in the regenerating plantlets was further confirmed using PCR. The kanamycin spraying test was used in the genetic segregation analysis^61^. We germinated the seeds of 14 T0 transgenic plants, and spray the kanamycin to identify the single locus plants based on the segregation rate (Supplementary Table S2). We kept the seeds of kanamycin-resistance green plants from three T1 transgenic lines (AnnSp2-18, 31 and 44) with the segregation of 3:1 based on chi square test. In T2 generation, we used the same method to screen the kanamycin-resistance green plants, and kept the T2 seeds from the plants without segregation, which indicated they are single locus homozygous lines. We confirmed that no segregation for them in T3 generation, and named them as OE1, OE2 and OE3 for convenience in following experiment.

### Expression analysis by qRT-PCR

Total RNA was extracted using Trizol reagent (Invitrogen) according to the manufacturer’s protocol. Quantitative RT-PCR was performed on a LightCycler 480 system (Roche, Switzerland). The reaction mixture contained 5 µl of 2 × SYBR premix Ex Taq (TAKARA), 0.5 µM each of forward and reverse primers, and 1 µl of 10-fold diluted first strand cDNA. The PCR program was as follows: pre-denaturation at 95 °C for 30 s; 40 cycles of 95 °C for 30 s, 53 °C for 15 s and 72 °C for 15 s; and a melt cycle from 65 to 95 °C. The tomato β-actin gene (GenBank accession No. BT013524) was used as an internal control. The primer sequences are listed in Supplementary Table S1. The threshold cycle value was given by the program automatically. The gene expression data were analyzed using the ^ΔΔ^Ct method^62^.

### Protein extraction and western blot analysis

Proteins were extracted using a modified trichloroacetic acid/acetone procedure as described by ref.^1^. For western blot analysis, Proteins were separated on 12% SDS-polyacrylamide gels, transferred onto nitrocellulose membranes and incubated with appropriate anti-AnnSp2 antisera overnight at 4 °C. Antibody-bound proteins were detected with secondary antibody conjugated to horseradish peroxidase, and visualized using an ECL system (Amersham Biosciences).

### GFP fusion construct and transient expression assay

To detect the subcellular localization of AnnSp2, an AnnSp2-GFP fusion vector was constructed. The full-length ORF of AnnSp2 without stop codon was amplified by PCR using a pair of primers (Supplementary Table S1) containing the X*ba1* and X*ho1* restriction enzyme sites (underlined) in the forward and reverse primers, respectively. Then, we digested the modified pUC18 vector fused with GFP with X*ba1* and X*ho1* and ligated the AnnSp2 gene to the vector. The gene expression was driven by the cauliflower mosaic virus (CaMV) 35 S promoter. The resulting 35S-AnnSp2-GFP fusion product was verified by double digestion and sequencing. The plasmid carrying the AnnSp2-GFP was co-expressed with NLS-RFP in *N. benthamiana* protoplasts for 12 h and the images were taken with a confocal microscope (Zeiss LSM510 META, Germany). NLS-RFP is a control for nuclear localization.

### Stress tolerance assays

To investigate the function of AnnSp2 in stress tolerance, stress assays were conducted at the germination and adult-plant stages on three transgenic lines overexpressing (OE1, OE2 and OE3) and one wild-type (WT) line. For the germination assay, the seeds were surface-sterilized and planted on MS medium containing different concentrations of abscisic acid (ABA), mannitol (300 mM), or NaCl (150 mM), and the germination rate was measured daily. For the stress tolerance assay, the seeds were germinated on MS medium for 3 d. When the radicle emerged, the plants were transferred to the medium supplemented with either ABA (1, 5 to 10 μM), mannitol (300 mM), or NaCl (200 mM) and placed upright in the chamber. The seedling length and weight was measured after the treatments.

For the drought treatment, plants were grown in a pot with sufficient water for 4 weeks, and then the water was withheld to subject the plants to drought stress for the duration indicated. Plants were left unwatered for 14d and then photographed, and the survival rate was recorded after re-watering for 1 week. To measure water loss, fully expanded leaves of the WT and transgenic lines were detached from 4-week-old plants and placed on filter paper under white florescent light and weighed at the indicated time intervals. The rate of water loss was calculated relative to the initial fresh weights.

For the salt stress treatment, soil-grown plants were treated with 200 mM NaCl (200 ml) every day and incubated under the same growth conditions as described above. The chlorophyll (Chl) content was analyzed according to ref.^63^. To observe the ABA response, a stomatal aperture assay was performed essentially as previously described^64^. Epidermal peels from the leaves of transgenic and WT plants treated with1 µM ABA for 3 h to assess the stomatal response. The ratio of stomatal length to width indicated the degree of stomatal closure. At least 50 stomatal apertures were measured for each line.

### Oxidative stress tolerance assay in transgenic tomato plants

For oxidative stress treatment, sensitivity analysis to methyl viologen (MV) was carried out during the vegetative stage. Leaf discs (1.3 cm in diameter) were suspended in solutions of various concentrations of MV (0, 1 and 3 µM) for 3 days. The differences between the transgenic lines and control were observed after chlorophyll content was measured^65^. The experiment was repeated twice.

### Determination of ABA and proline contents

For ABA quantification, ABA was extracted from 0.5 g of 2-week-old seedlings as described by ref.^66^, and the endogenous ABA content was measured by ELISA according to the manufacturer’s instructions. Proline was extracted from 0.2 g of 2-week-old seedlings and quantified by the ninhydr in method using the procedure described by ref.^67^.

### Histochemical detection of ROS and measurement of MDA content

A histochemical staining procedure was used to detect the accumulation of H_2_O_2_ and O^2−^ using 3,3ʹ diaminobenzidine (DAB). Detached leaves were incubated in DAB (1 mg/ml, pH 3.8) overnight at room temperature in the dark. The stained leaves were then boiled in 95% ethanol to remove the chlorophyll (Chl) before imaging. MDA was estimated for indirect evaluation of lipid peroxidation using thiobarbituric acid as described by ref.^68^.

### Antioxidant enzyme activity assays

The antioxidant enzyme activities of superoxide dismutase (SOD), peroxidase (POD), catalase (CAT) and ascorbate peroxidase (APX) in the leaves were determined as previously described^13^. The leaves were homogenized in a blender in 2 mL of ice-cold 0.1 M phosphate buffer (pH 6.5), 2 mM ascorbic acid, and 0.1% bovine serum albumin. The homogenate was filtered through six layers of cheesecloth and centrifuged at 4 °C for 15 min at 12,000 rpm. The pellet was suspended with 4 cm^3^ PBS for measurement of chloroplastic APX (peroxidase EC 1.11.1.7) and SOD (superoxide dismutase EC 1.15.1.1). Each assay was replicated at least three times per sample.

### Statistical analysis

The data were analyzed using least significant difference (LSD) and analysis of variance (ANOVA) using Statistical Analysis System (SAS) version 9.1 software. The results are expressed as the means ± SE of triplicate experiments (n = 3). Comparisons showing significant differences are shown.

## Electronic supplementary material

Supplementary Data
