# Supplementary material for: Overexpression of annexin gene AnnSp2, enhances drought and salt tolerance through modulation of ABA synthesis and scavenging ROS in tomato
Source: Sci Rep. 2017 Sep 21;7:12087. doi: 10.1038/s41598-017-11168-2 (PMC5608957; doi:10.1038/s41598-017-11168-2)
Supplement: Supplementary file 1 — Supplementary Data [file 41598_2017_11168_MOESM1_ESM.pdf]

RESEARCH ARTICLE

Overexpression of annexin gene AnnSp2, enhances drought and salt tolerance through modulation of ABA synthesis and scavenging ROS in tomato

Raina Ijaz[1], Javeria Ejaz[2], Shenghua Gao[1], Tengfei Liu[1], Muhammad Imtiaz[3], Zhibiao Ye[1,]*, Taotao Wang[1,]*

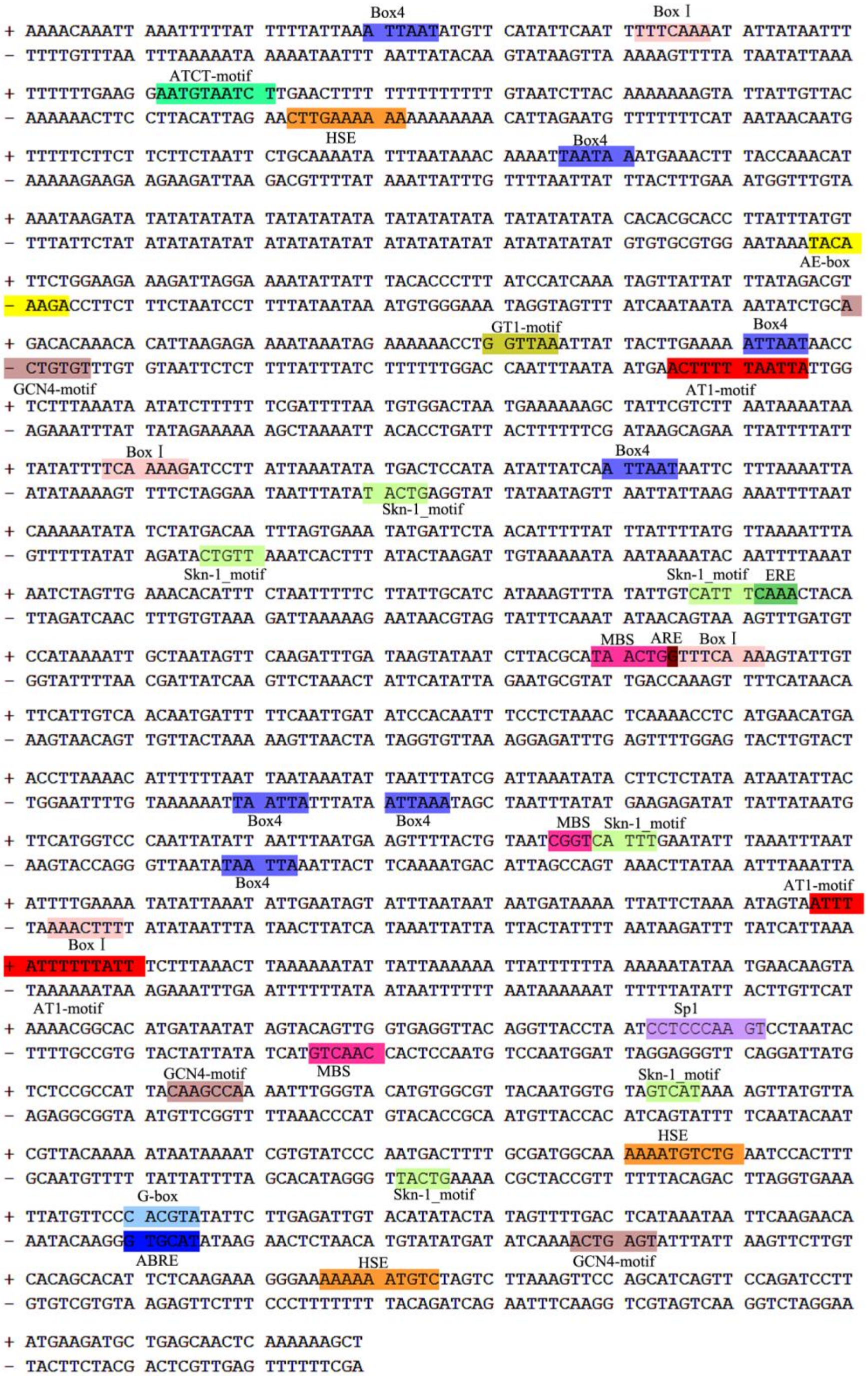

Fig. S1 *Cis*-acting element analysis of AnnSp2 promoter. ABRE (TACGTG), *cis*-acting element involved in abscisic acid responsiveness. ATCT-motif (AATCTAATCT), part of a conserved DNA module involved in light responsiveness. BOX 4 (ATTAAT), part of a conserved DNA module involved in light responsiveness. GCN4-motif (TGTGTCA), *cis*-acting regulatory element involved in endosperm expression. G-BOX (CACGTC/TACGTG), *cis*-acting regulatory element involved in light responsiveness. HSE (AAAAAATTTC), *cis*-acting element involved in heat stress responsiveness. SP1 (CC(G/A)CCC), light responsive element. TCA-element(GAGAAGAATA), *cis*-acting element involved in salicylic acid responsiveness. AT1-motif (ATTAATTTTACA/ AATTATTTTTTATT), part of a light responsive module.AE-box (AGAAACAT), part of a module for light response. GT1-motif (GGTTAA), light responsive element. TATC-box (TATCCCA), gibberellin-responsive element.MBS (TAACTG/CAACTG), MYB binding site involved in drought inducibility. ARE (TGGTTT), *cis*-acting regulatory element essential for the anaerobic induction.

A

B

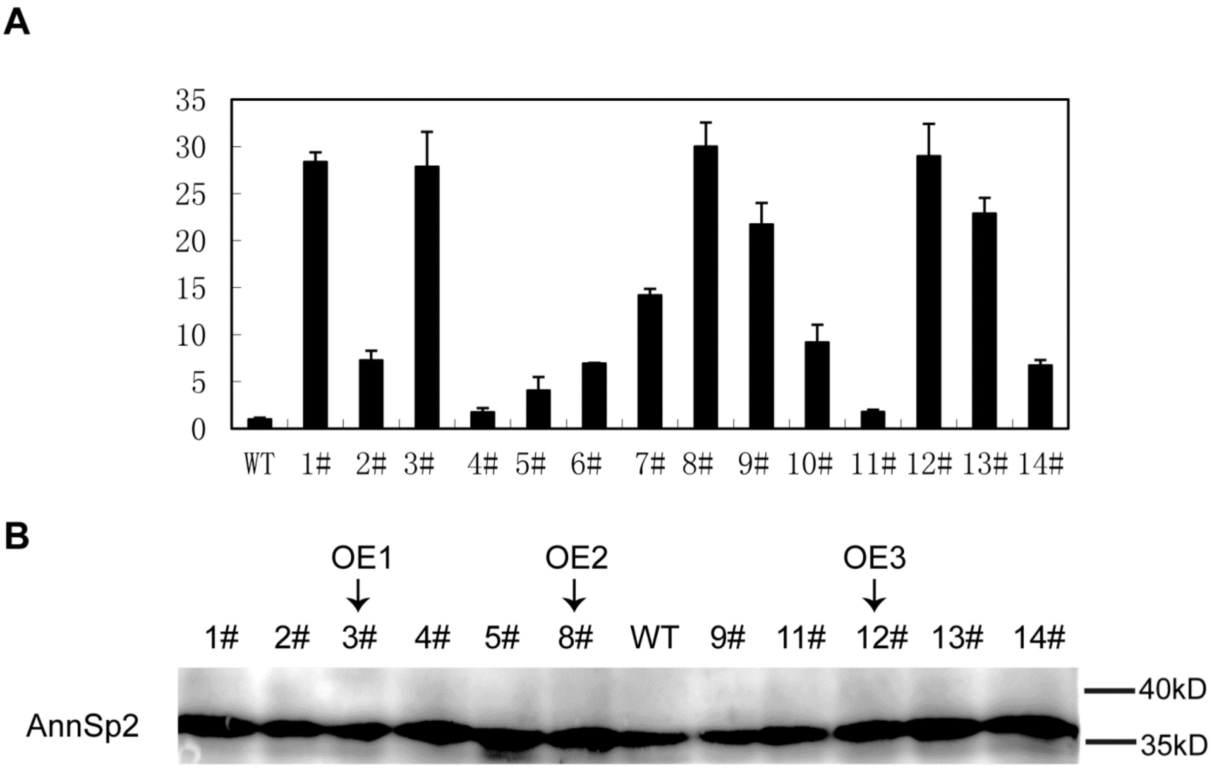

Fig. S2 A Identification of transgenic plants and expression of AnnSp2. (A) q-PCR analysis of AnnSp2 expression in wild-type (WT) and T2 OE plants. (B) Protein accumulation in AnnSp2. Western blot analysis of AnnSp2 transgenic plants and in wild-type (WT) plants.

Table S1 Details of the primers used in this study

Primer name	Forward primer sequence	Reverse primer sequence	Purpose
AnnSp2	TTCCCACGTATATTCTTGAGATTG	CACACCATACATCATAAAAGCAAA	Gene amplification
QAnnSp2	GATTCTTATGCTGCTGCTTATGGA	CAGTCAGACGTTTGGTAGCCTCA	Quantitative real-time-PCR primer
AnnSP2	CCGCTCGAGATGTCTAGTCTTAAAGTTCCA	TGCTCTAGATCAAGCATCTCCGTGCCCAATC	Subcellular localization primer
CaMV 35S	TTGAATCCTGTTGCCGGTCT	GGAAGGGTCTTGCGAAGGATAG	Transgenic plants confirmation
AREB	TTGCTGGTGGAAATGTAAGTGC	GGAATGTAACATCCTTTGAGTATCG	qRT-PCR
SPERD	AGGCATCAAGTCATCACTCTCTGGT	GAGTAATGTGAGTAAGAACCAACG	qRT-PCR
DREB	AGTTGGGGAAAATGGGTGTC	CGAGGCAATGAGTCAATTAGGT	qRT-PCR
NCED	AGTTTCCCGATTTGGTATTC	TGGAGTCTGGTGGTGTCA	qRT-PCR
P5CS	GCGATTCCTGGAACTGTTGG	AGCATGGCCAAGAACAGGAA	qRT-PCR
β-actin	ACCTTCAATGTCCCTGCTATG	CTCCACCTTCAGAAACGCAAC	β-actin

Table S2 The genetic segregation analysis of over expression AnnSp2 plants in T1 generation by spraying kanamycin test

**Number**	**Gene Name**	**T1 segregation**		**Single locus with 3:1 segregation***
		**Green**	**yellow**	
1	AnnSp2 -6	18	3	No
2	AnnSp2 -14	32	7	No
3	AnnSp2 -18	29	10	Yes
4	AnnSp2 -20	33	16	No
5	AnnSp2 -26	44	12	No
6	AnnSp2 -27	21	5	No
7	AnnSp2 -29	27	13	No
8	AnnSp2 -31	36	12	Yes
9	AnnSp2 -36	15	6	Yes
10	AnnSp2 -38	15	9	No
11	AnnSp2 -42	14	2	No
12	AnnSp2 -44	12	4	Yes
13	AnnSp2 -50	23	16	No
14	AnnSp2 -56	17	4	No

* The analysis based on chi square test
